# Etiology of Hypopituitarism in Adult Patients: The Experience of a Single Center Database in the Serbian Population

**DOI:** 10.1155/2017/6969286

**Published:** 2017-06-18

**Authors:** M. Doknić, S. Pekić, D. Miljić, I. Soldatović, V. Popović, M. Stojanović, M. Petakov

**Affiliations:** ^1^Neuroendocrine Department, Clinic of Endocrinology, Diabetes and Metabolic Diseases, Clinical Center of Serbia, Belgrade, Serbia; ^2^School of Medicine, University of Belgrade, Belgrade, Serbia; ^3^Institute of Medical Statistics and Informatics, Belgrade, Serbia

## Abstract

There are only a few published studies related to the population-based etiology of hypopituitarism. New risks for developing hypopituitarism have been recognized in the last 10 years. *Aim*. To present data regarding the etiology of hypopituitarism collected in a tertiary center over the last decade. This is a cross-sectional database study. *Patients and Methods*. We included 512 patients (pts) with hypopituitarism, with a mean age of 45.9 ± 1.7 yrs (range: 18–82; male: 57.9%). *Results*. Nonfunctional pituitary adenomas were presented in 205 pts (40.5%), congenital causes in 74 pts (14.6%), while acromegaly and prolactinomas were presented in 37 (7.2%) and 36 (7.0%) patients, respectively. Craniopharyngiomas were detected in 30 pts (5.9%), and head trauma due to trauma brain injury-TBI and subarachnoid hemorrhage-SAH in 27 pts (5.4%). Survivors of hemorrhagic fever with renal syndrome (HFRS) and those with previous cranial irradiation were presented in the same frequency (18 pts, 3.5% each). *Conclusion*. The most common causes of hypopituitarism in our database are pituitary adenomas. Increased awareness of the other causes of pituitary dysfunction, such as congenital, head trauma, extrapituitary cranial irradiation, and infections, is the reason for a higher frequency of these etiologies of hypopituitarism in the presented database.

## 1. Introduction

Hypopituitarism is defined as the partial or complete loss of anterior pituitary function that can result from acquired or congenital causes. According to previous large surveillance databases, hypothalamo/pituitary tumors and their attendant treatments, including surgery and radiotherapy, account for approximately 70% of the acquired causes of pituitary insufficiency [1–3]. However, with increasing knowledge about the etiology of hypopituitarism, nontumoral causes are becoming more evident [4]. In the recent years, new gene mutations affecting hypothalamo/pituitary embryogenesis have been revealed, and novel genetic causes of congenital hypopituitarism [5, 6] have been identified.

Traumatic brain injury-TBI, subarachnoid hemorrhage, and extrapituitary cranial irradiation have also been recognized as the causes of hypopituitarism in the last decade [7, 8]. Until recently, TBI has been considered a rare cause of hypopituitarism. In our previous study, hypopituitarism caused by TBI was reported in one-third of the investigated patients [9]. Later investigations have detected pituitary dysfunction following TBI in approximately 25% of the patients [10, 11]. On the other hand, recently published studies suggest that the actual rate of TBI-related hypopituitarism may not be as high as previously thought [12, 13]. A recent review has shown that the rate of hypopituitarism after TBI may vary from negligible to over 50% of patients, reflecting probably methodological differences in terms of patient selection, study designs, and diagnostic procedures [14].

Subarachnoid hemorrhage (SAH) has emerged as another recently identified risk factor for hypopituitarism, particularly growth hormone and corticotrophin deficiencies [15]. Conducted studies provide evidence for the prevalence of neuroendocrine dysfunction in SAH survivors, ranging from 0 to 55% [16]. A recently published study has shown that pituitary dysfunction as a late complication of SAH is present in 25% of patients [17]. Neuroendocrine disturbances may develop or improve in months, or even three years, after TBI and SAH. Because of a possible recovery of the pituitary function as well as new onset hypopituitarism after these two causes of head trauma, follow-up testing is advisable.

Patients, who received radiotherapy for brain and neck tumors, or whole-body irradiation for hematological malignancies in childhood, risk the loss of pituitary function later in life [18, 19]. About 40% of the patients irradiated for brain tumors distant from the hypothalamo/pituitary region developed hypopituitarism [20, 21]. Pituitary deficit in one or more axes may occur two to ten years after irradiation [22].

The recognition of infectious/inflammatory diseases as the important causes of hypopituitarism has increased in the recent years [23]. Pituitary abscesses and the infection of the central nervous system of various etiologies, tuberculosis-TBC, and other bacterial, viral, and fungal infections can affect hypothalamo/pituitary axis and disrupt its function [24, 25]. The importance of early diagnosis of these rare alternative etiologies of hypopituitarism has been emphasized [26, 27]. Unrecognized hypopituitarism may be misdiagnosed as persistent neurologic sequel or cognitive impairment in TBI; pituitary abscess can be misinterpreted as a pituitary adenoma, while pituitary insufficiency following meningoencephalitis may be misunderstood as postencephalitic syndrome. Therefore, it is of great importance to raise the awareness of the alternative and less common causes of hypopituitarism among medical specialties dealing with these patients.

Adults suffering from hypopituitarism have an increased morbidity and mortality rate compared with that of the general population, mostly due to cardiovascular diseases [28, 29]. Early diagnosis is important to mitigate the consequences of hypopituitarism and extend a patient's lifespan. However, the clinical manifestations of pituitary dysfunction are subtle and nonspecific in most patients, which is why the diagnosis is often delayed.

Even though the origin of hypopituitarism is the subject of numerous articles, there are only a few published studies related to population-based etiology of hypopituitarism [30–32]. The aim of this study is to present the causes of hypopituitarism in the Serbian population based on the data collected in a single reference center over the last decade, when new alternative causes of hypopituitarism started to emerge as relevant for adult endocrinologists.

## 2. Patients and Methods

This cross-sectional database study was conducted at the Neuroendocrine Department at the Clinic for Endocrinology, Diabetes and Metabolic Diseases, the Clinical Center of Serbia from January 2005 to June 2016. We have enrolled 512 adult patients with hypopituitarism attending our department during this period of time. In this retrospective population study that lasted almost 12 years, we collected patient data regarding their sex, age, auxological characteristics, the precise causes of pituitary dysfunction, age at the time of hypopituitarism diagnosis, duration of hypopituitarism, number and type of deficient pituitary hormones, diagnostic test for pituitary failure, data on surgical and radiation therapy, hormonal replacement, cranial MR imaging, and comorbidities. The inclusion criteria were (1) age over 18 years, (2) hypopituitarism diagnosed at birth, or acquired during childhood or adult life, and (3) deficiency of one or more pituitary hormones, including the antidiuretic hormone (ADH). Patients with unreliable or missing data were excluded from the analysis in this study. Our patients were monitored every 6 to 12 months in terms of adequacy of hormonal therapy, comorbidities, and successive MRIs of the hypothalamo/pituitary region.

A group of patients with congenital hypopituitarism consisted of adults with structural abnormalities of the stalk and pituitary gland, such as hypoplastic pituitary, pituitary stalk disconnection, ectopic posterior pituitary, septo-optic dysplasia, and congenital hypogonadotropic hypogonadism; patients with confirmed PROP 1 mutation, and siblings with familial hypopituitarism. Almost all of them were directed to us from our transition clinic when they turned 18.

In the group of patients with head trauma, we included adults who suffered from traumatic brain injury-TBI and subarachnoid hemorrhage (SAH). Patients with pituitary insufficiency, as a late-onset sequel of extrapituitary cranial irradiation for intracerebral and nasopharyngeal tumors, or total body radiotherapy for hematological malignancies during childhood were also enrolled in our investigation.

The diagnosis of permanent partial or complete hypopituitarism was confirmed according to the standard diagnostic criteria [33–35]. Anterior and posterior pituitary insufficiency was established in a clinical context, based on the basal hormonal levels and stimulation tests.

Serum samples for free thyroxine (FT4), thyroxine (T4), thyroid-stimulating hormone (TSH), follicle-stimulating hormone (FSH), luteinizing hormone (LH), prolactin (PRL), cortisol, and insulin-like growth factor I (IGF-I) determination were taken after an overnight fast at 08:00 AM. Serum total testosterone was determined in all males, and serum estradiol in females. A menstrual history was taken into account in all female subjects. Hormones were measured by the following commercial kits: T4 by RIA (INEP, Zemun, Serbia); TSH by IRMA (INEP, Zemun, Serbia); and PRL, LH, and FSH by IRMA (Cis Bio International, France). Cortisol, FT4, testosterone, and estradiol were measured by RIA (Cis Bio International, France). IGF-I was measured by a chemiluminescent enzyme immunoassay with the Immulite Analyzer (Diagnostic Product Corporation, Los Angeles, CA, USA). Diabetes insipidus was suspected in the patients with polyuria (>3 L/day) of dilute urine (<300 mOsm/kg). When these measurements did not enable a valid conclusion, appropriate provocative tests were performed (insulin tolerance test (ITT), GH-releasing hormone (GHRH)/GH releasing peptide-6 (GHRP-6) test, glucagon stimulation test, and adrenocorticotropic hormone (ACTH) stimulation test (low dose 1 *μ*g) for the assessment of secondary hypocorticism in patients where ITT was contraindicated, luteinizing hormone-releasing hormone (LHRH) test, and water deprivation test).

In determining the cause of pituitary hormone deficiency in our patients, pituitary and cranium MRI were performed. Histological analyses of the removed pituitary tumors or sellar masses were available for all the operated patients. The majority of patients were examined as in-patients at our center. The patients were sent to us by pediatricians, surgeons and specialists of internal medicine from primary, secondary, and tertiary care institutions.

The results were analyzed by using the SPSS 20.0 program and expressed as mean ± standard error of the mean (mean ± SE) and in percentages (%). Group comparisons were performed using the chi-square test, Kruskal-Wallis test, and Mann–Whitney *U* test, depending on data type. All *p* values less than 0.05 were considered significant.

## 3. Results

We included 512 adult patients with confirmed hypopituitarism and a mean age of 45.9 ± 1.7 yrs (range 18–82). The mean age did not differ by sex, 46.4 ± 16.8 yrs (range: 18–79) for female patients and 45.5 ± 17.1 yrs (range: 18–82) for male patients (*p* > 0.05). Hypopituitary males account for 57.9% of our database (*n* = 297), and females for 42.1% (*n* = 215). The mean age at the diagnosis of hypopituitarism was 39.8 ± 22.3 yrs. Adult onset of hypopituitarism (AOH) was reported in 75.2% pts (*n* = 385), and childhood onset of hypopituitarism (COH) in 24.8% pts (*n* = 127).

The etiology of hypopituitarism is shown in Table 1. Most of our patients (338 patients; 65.8%) had suffered from tumor or cyst in the hypothalamo/pituitary region. The total number of patients with pituitary adenomas that were confirmed histologically was 288 (56.1%). The five most common causes of hypopituitarism in our database were nonfunctioning pituitary adenomas (205 patients; 40.5%), followed by congenital causes (74 patients; 14.6%), acromegaly (37 patients, 7.2%), prolactinomas (36 patients, 7.0%), and craniopharyngioma (30 patients; 5.9%). Head trauma (TBI and SAH) frequency was presented in a slightly lower percentage (27 patients; 5.4%). Although viral infections rarely cause hypopituitarism, we recorded 18 hypopituitary patients (3.5%), who had survived hemorrhagic fever with renal syndrome (HFRS) caused by Hanta viruses. Pituitary dysfunction as a late consequence of extrapituitary cranial irradiation was determined in the same frequency (18 patients, 3.5%). Other causes of anterior pituitary deficiency are reported in less than 3% in each category (Table 1).

We analyzed the characteristics of patients with the most common causes of hypopituitarism in our population (Table 2). As we expected, patients with congenital causes of pituitary insufficiency and those with cranial irradiation were significantly younger than the patients from other groups (*p* < 0.001). Regarding distribution by gender, similar percentages of the sexes were reported in all patient groups, except for prolactinomas and HFRS survivors, where males are predominant (72.2% and 100%, resp.). Complete hypopituitarism was most prevalent in patients with craniopharyngioma and pituitary tumors, whereas those with a history of TBI and SAH usually had only one hormone deficit (*p* < 0.001).

Out of the total number of enrolled patients, 246 (57.2%) underwent neurosurgery, while 68 (16.3%) received radiotherapy.

**Figure 1:**
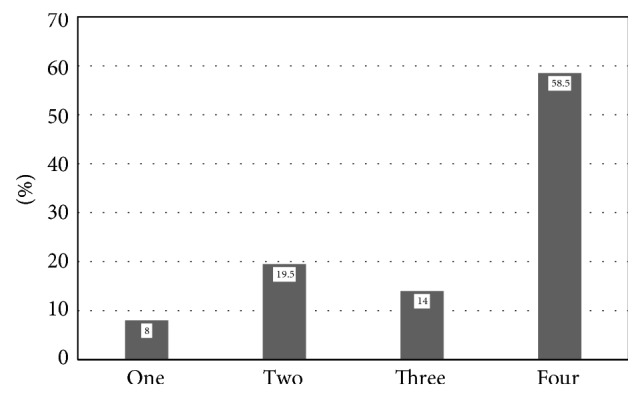
Prevalence of loss of four, three, two, and one anterior pituitary axis.

The majority of patients included in our database had multiple pituitary hormone deficiencies. Two hundred and eighty-five patients (58.5%) had all four pituitary axes affected, 68 patients (14.0%) had three, 95 (19.5%) had two, and 39 patients (8.0%) had one disturbed pituitary axis (Figure 1).

**Figure 2:**
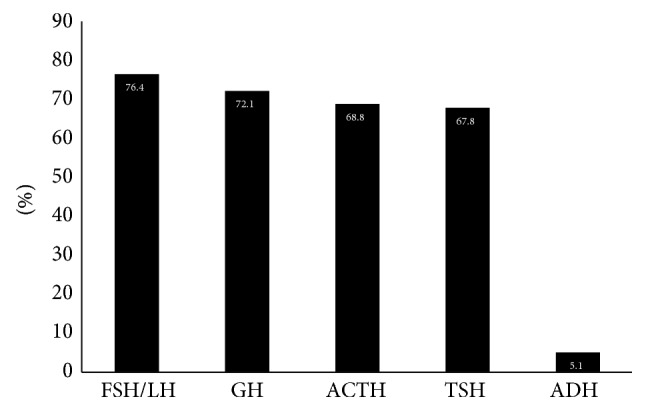
Frequency of pituitary hormone deficiencies.

Gonadotrophins (FSH/LH) and growth hormone (GH) deficiency are the most common pituitary hormone deficiencies, 76.4% (391 patients) and 72.1% (369 patients), respectively, followed by ACTH (352 patients, 68.8%) and TSH (346 patients, 67.8%) deficiencies (Figure 2). Posterior pituitary insufficiency was recorded in 26 patients (5.1%).

**Figure 3:**
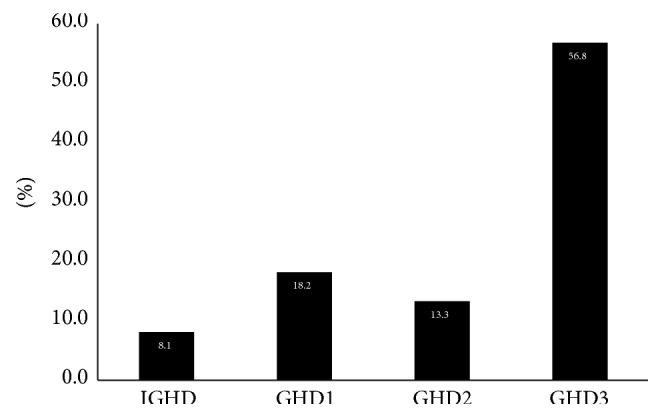
Number of anterior pituitary hormones deficiencies associated with GHD (IGHD—isolated growth hormone deficiency (8.1%); GHD1—growth hormone deficiency plus one more deficient anterior pituitary hormone (18.2%), GHD2—growth hormone deficiency plus two more deficient anterior pituitary hormones (13.3%), and GHD3—growth hormone deficiency plus three more deficient anterior pituitary hormones (56.8%) patients).

Generally, the growth hormone is the first hormone to be affected, which is why we were interested in the deficiency of the growth hormone (GHD) in combination with the deficiencies of other pituitary hormones. Thus, the loss of GHD was reported as well as the loss of another one, two, or three pituitary hormones in 91 (18.2%), 66 (13.3%), and 284 patients (56.8%), respectively. An isolated growth hormone deficiency (IGHD) was recorded in 28 patients (8.1%) (Figure 3).

## 4. Discussion

This study shows a wide spectrum of different causes of hypopituitarism in the Serbian population. We have presented the experience of a single tertiary referral center. Our database enrolled 512 patients with confirmed partial or complete pituitary insufficiency. In accordance with other studies, hypothalamo/pituitary tumors are the leading etiological factors of pituitary insufficiency in our database (65.8%). We have confirmed that the most common cause of hypopituitarism is a nonfunctioning pituitary adenoma (40.5%), followed by congenital causes (14.6%), prolactinomas and GH-secreting adenomas equally (7.0% and 7.2%), and craniopharyngiomas (5.9%). However, in contrast to other studies, nontumoral causes, such as congenital hypopituitarism and trauma brain injury, are present in a significant percentage in our study. A higher frequency of congenital hypopituitarism in this study is influenced by the raised awareness of our pediatric endocrinologists regarding pituitary insufficiency during the transition period and the referral of these patients in the last decade.

The higher frequency of patients with traumatic brain injury, SAH, or cranial irradiation in our database is a consequence of our specific interests in these causes of hypopituitarism during the last decade. In collaboration with neurosurgeons, neurologists, and neuroradiologists, we performed several studies focused on the detection of these etiological factors of hypopituitarism in our population [9, 15, 21].

After an epidemic of hemorrhagic fever with renal syndrome (HFRS) caused by the Hantavirus in Serbia 15 years ago, in cooperation with our nephrology department, we tested 60 adults who survived HFRS and diagnosed hypopituitarism in 8 patients [36]. It is manifested by a severe systemic infection, with acute shock, acute renal failure, and pituitary ischemia/infarction. Pituitary dysfunction, as a late complication of HFRS, often remains unrecognized due to subtle clinical manifestations. HFRS is endemic in certain regions of the Balkans and in some war zones. Rodents are the carriers of Hantavirus; therefore, soldiers and farmers are at increased risk of this infection. The presented database contains a noticeable percentage of this rare etiological factor of hypopituitarism (3.5%).

Data pertaining to the causes of the pituitary hormone deficiencies specific for the country population are sparse. The largest surveillance database regarding population-based characteristics of hypopituitary patients was conducted by the Dutch National Registry, which included nearly 2900 GHD adults over a period of 10 years [30]. In almost one-third of the total group of patients, pituitary dysfunction was caused by a nonsecreting pituitary adenoma, followed by a pituitary-secreting adenoma (16%), craniopharyngioma (11%), and idiopathic hypopituitarism (7%). Interestingly, in their database, ACTH-producing adenomas were the most prevalent in the group of pituitary-secreting adenomas with GHD. The frequency of congenital causes was higher in our study, while we recorded craniopharyngioma in a lower percentage compared to the Dutch data.

Tanriverdi et al. presented a study related to the etiology of hypopituitarism in the Turkish population, by analyzing 773 hypopituitary adult patients [31]. That study has shown that nontumoral disorders were the most common causes of pituitary dysfunction among all the patients (43%). When analyzed by gender, the nonfunctioning pituitary tumor was the most common cause of the pituitary dysfunction in males (15%), while in females, it was Sheehan's syndrome (14%).

In a retrospective population study from the northwestern region of Spain, a series of 209 hypopituitary adult patients was evaluated [32]. In the majority of the included patients, the diagnosis of pituitary or peripituitary tumor was established (55%), and nearly half of these suffered from nonfunctioning pituitary adenomas, which is consistent with our results. However, our study has demonstrated a lower prevalence of pituitary-secreting adenoma in comparison to the Spanish study, in which acromegaly, prolactinomas, and Cushing's disease were reported in 25%, 13%, and 7%, respectively.

The etiology of hypopituitarism can largely vary between different countries. The prevalence of some very rare causes is greater in the countries situated in the tropics than in the developed countries. In these regions, the infective causes of hypopituitarism, such as pituitary abscess, HIV infection, and tuberculosis, are not rare in comparison with the developed countries [37]. In countries with a lower level of obstetric care, Sheehan's syndrome is the leading cause of hypopituitarism in females, while in developed countries, it is very rarely reported [38]. Acute and chronic hypopituitarism following snakebite have been reported in some Asian countries [39]. A study conducted at a tertiary care hospital in India recorded pituitary adenoma in 38%, while Sheehan's syndrome in 27%, and the snakebite in 15% of adult hypopituitary patients [40]. On the other hand, in developed countries, new modalities of anticancer therapy were published as case reports of pituitary insufficiency. Autoimmune lymphocytic hypophysitis with hypopituitarism secondary to ipilimumab therapy has been reported in patients treated with this antitumor monoclonal antibody [41].

A different prevalence of the causes of hypopituitarism, even among developed countries, has been reported [42]. Thus, the pituitary adenomas and associated treatments in the West European (WE) countries ranged from 45 to 65% of the evaluated patients; in the US, this etiologic factor of pituitary deficiency was documented in only 20%. Also, idiopathic growth hormone deficiency was dominant in the US database (57%), while it accounts for only 9% in the WE countries. On the other hand, regarding ethnic differences, a recent study has shown that the etiology of hypopituitarism in 349 Japanese hypopituitary adults is similar to that of the Caucasian population [43].

There is a noticeable difference in the prevalence of certain causes of hypopituitarism today compared to a few decades ago. During the last ten years, reports have shed light on many previously unrecognized causes of pituitary insufficiency [44–46]. Brabant et al. have analyzed the etiology of hypopituitarism in more than 13,000 patients from 30 different countries within the KIMS database [42]. The great majority of the patients had growth hormone deficiency due to pituitary adenoma (44%) and craniopharyngioma (11%), whereas other causes, such as brain tumor irradiation and TBI, were confirmed in only 7% and 3% of the patients, respectively. A study published several years earlier also reported a higher frequency of the hypothalamic and pituitary tumors and lower incidence of brain radiation and TBI in the KIMS database patients [47]. According to another longitudinal global surveillance database, there is an obvious decline in the proportion of pituitary adenoma, craniopharyngioma, and the pituitary postpartum necrosis, with an increasing frequency of the less common causes of hypopituitarism in adults [48].

Idiopathic hypopituitarism (IH) means that the cause of pituitary hormone deficiency cannot be identified. An idiopathic cause is reported in about 10–15% of the patients with adult GHD in global surveillance studies [49]. Wilson et al. reported that in 230 patients with hypopituitarism, 21 (9%) had normal pituitary imaging and no identifiable cause of pituitary hormone deficiency [50]. In the majority of the investigated patients, careful and detailed consideration of the clinical history was helpful in finding the cause of hypopituitarism. Thus, a history of TBI, neurosarcoidosis, hemochromatosis, meningitis, and SAH was detected in that group of patients. Our study reported only 2 patients with idiopathic adult onset hypopituitarism. Both patients had complete anterior pituitary insufficiency. After excluding all the reasons known to us for pituitary insufficiency, we have considered the pituitary apoplexy and the disappearance of previous pituitary adenoma, or the presence of a high titer of antipituitary antibodies [51]. The genetic causes of idiopathic hypopituitarism are rare [52, 53]. An overall rate of 8% of mutations was found in sporadic idiopathic hypopituitarism, while familial cases with IH are highly suspected of genetic causes [54].

Regarding the type of hormonal deficiency, we showed that FSH/LH was the most frequent (76.4%), followed by GH (72.1%), ACTH (68.8%), and TSH (67.8%) deficiencies. Our results are in agreement with the results of other studies, where gonadotrophins and GH axes are the most commonly affected [25]. In the KIMS database, where all the patients have GHD, gonadotrophin deficiency was confirmed in 70%, TSH in 68%, and ACTH in 61% of them [42].

Considering the number of deficient hormones, the presented study demonstrated that around 70% of the patients had a loss of 3-4 pituitary hormones. Our data compared to those of other studies show a more pronounced hypopituitarism [30–32].

## 5. Conclusion

The etiology of hypopituitarism may depend on the geographic region and time when the investigation was conducted. Similar to other studies, we have shown that the main cause of the pituitary insufficiency is hypothalamo/pituitary tumor. It is followed by the frequency of nontumoral causes, such as congenital hypopituitarism and head trauma. Our transition clinic with pediatric endocrinologists, in the past years, influenced the presence of such a high percentage of congenital hypopituitarism. Moreover, our recent studies focusing on patients with head trauma, cranial irradiation, and infections are the reason for a higher frequency of these causes of hypopituitarism in our database.

Raising awareness of the possible causes of hypopituitarism in other specialties of medicine apart from endocrinology is important, because unrecognized pituitary dysfunction significantly affects the physical and psychological well-being. The newly discovered cryptic causes of hypopituitarism narrow the number of unclear causes of hypopituitarism.

## Figures and Tables

**Table 1:** Etiology of hypopituitarism in all enrolled patients (*N* = 512).

		No.	%
Congenital hypopituitarism		74	(14.6)
Head trauma	Trauma brain injury	20	(4.0)
	Subarachnoid hemorrhage	7	(1.4)
	Extrapituitary cranial irradiation	18	(3.5)
Pituitary tumors	Acromegaly and gigantism	37	(7.2)
	Prolactinomas	36	(7.0)
	Nonfunctional pituitary tumors	205	(40.5)
	Cushing's disease	10	(2.0)
	Pituitary cyst	9	(1.8)
Tumors of sellar region	Craniopharyngioma	30	(5.9)
	Germinoma	7	(1.4)
	Meningioma, chondroma, granulosa cell tumor	4	(0.8)
	Metastasis	2	(0.4)
Infections	Viral	18	(3.5)
	Tuberculosis	1	(0.2)
	Fungal	2	(0.4)
Vascular disorders	Sheehan's syndrome	6	(1.2)
	Pituitary apoplexy	2	(0.4)
	Aneurysm	3	(0.6)
Inflammations/infiltrations	Lymphocytic hypophysitis	8	(1.6)
	Histiocytosis X	3	(0.6)
Empty sella		6	(1.2)
Other	^•^CHARGE Sy, ^••^Leukodistropy 4H Sy	2	(0.4)
Idiopathic		2	(0.2)

^•^CHARGE Sy includes coloboma of the eye, heart defects, atresia of the choanae, retardation of growth and/or development, genital abnormalities, and ear abnormalities.

^••^Leukodystrophy 4H Sy includes hypomyelination, hypogonadotropic hypogonadism, and dental developmental anomalies.

**Table 2:** Characteristics of the most common causes of hypopituitarism in our database (TBI—trauma brain injury; SAH—subarachnoid hemorrhage; HFRS—hemorrhagic fever with renal syndrome caused by Hantavirus).

Cause of hypopituitarism	Age (mean ± SE)	Sex, males (N/%)	Number of deficient hormones (mean ± SE)
Nonfunctional adenoma	56.1 ± 0.9	123/59.1	2.5 ± 0.6
Congenital causes	30.2 ± 1.4	43/57.3	2.3 ± 0.1
GH adenoma	45.3 ± 2.3	21/56.8	2.3 ± 0.1
PRL adenoma	49.7 ± 2.0	26/72.2	1.8 ± 0.2
Craniopharyngioma	36.0 ± 2.6	17/56.7	2.7 ± 0.1
TBI + SAH	48.2 ± 2.7	16/59.3	1.1 ± 0.2
Extrapituitary cranial irradiation	28.6 ± 2.8	11/61.1	1.6 ± 0.1
HFRS	35.1 ± 2.0	18/100	1.5 ± 0.2
